# Supplementary material for: Strain-shear coupling in bilayer MoS2
Source: Nat Commun. 2017 Nov 8;8:1370. doi: 10.1038/s41467-017-01487-3 (PMC5678169; doi:10.1038/s41467-017-01487-3)
Supplement: Supplementary file 1 — Supplementary Information [file 41467_2017_1487_MOESM1_ESM.pdf]

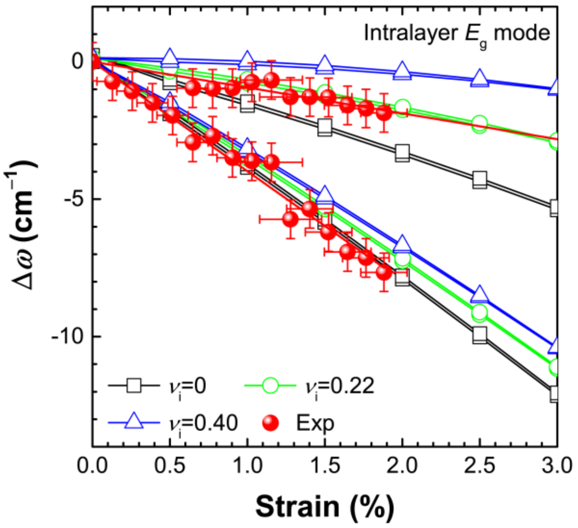


**Supplementary Figure 1| Dependence of shift rates of intralayer $E_g$ modes on Poisson's ratio.** Comparison of shift rates of intralayer $E_g$ modes calculated for three different in-plane Poisson's ratio values of 0 (black square), 0.22 (green circle, intrinsic value of $MoS_2$), and 0.40 (blue triangle, value for acrylic substrate). Experimental results are shown as red symbols. All the results are for $\varphi$ =15.7°. Experimental error bars in strain come from the uncertainty in the estimate of the strain from the curvature of the bent substrate. The error bars in $\Delta\omega$ are defined by the standard deviation of experimental values.

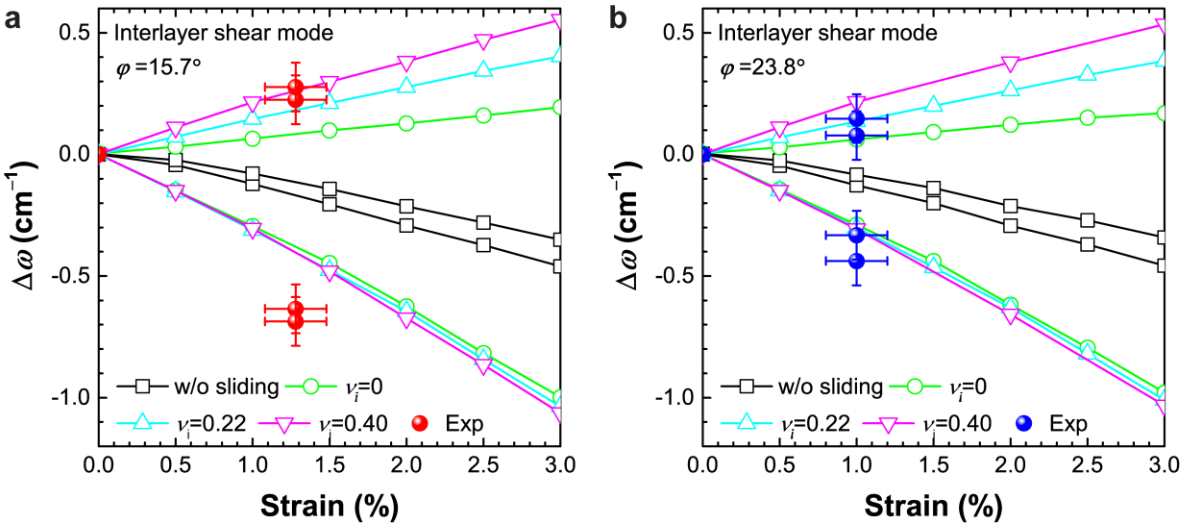


**Supplementary Figure 2| Effect of sliding on peak shift of interlayer shear mode as a function of uniaxial strain.** Calculated peak shifts without including sliding (black square) and with sliding (other open symbols) are shown for (**a**) $\varphi$ =15.7° and (**b**) $\varphi$ =23.8°. Peak shifts with sliding are estimated for three different Poisson's ratio values of 0 (green circle), 0.22 (cyan triangle, intrinsic value of $MoS_2$), and 0.40 (magenta reversed triangle, value for acrylic substrate). Experimental results as filled symbols (red for $\varphi$=15.7° and blue for $\varphi$ =23.8°) are also shown. Experimental error bars in strain come from the uncertainty in the estimate of the strain from the curvature of the bent substrate. The error bars in $\Delta\omega$ are defined by the standard deviation of experimental values.

**Supplementary Note 1: Calculation of peak splitting based on ball-and-stick model**

The frequencies of the interlayer shear phonon modes in the Raman spectrum are determined by the effective interatomic spring constants between bottom sulfur atoms of the upper $MoS_2$ layer and the top sulfur atoms of the lower layer as in Supplementary Figs. 3a and b. Supplementary Fig. 3c shows the top view schematically with the sulfur atom from the upper layer having lighter color. The parameters $k_1$, $k_2$, and $k_3$ are the lateral components of

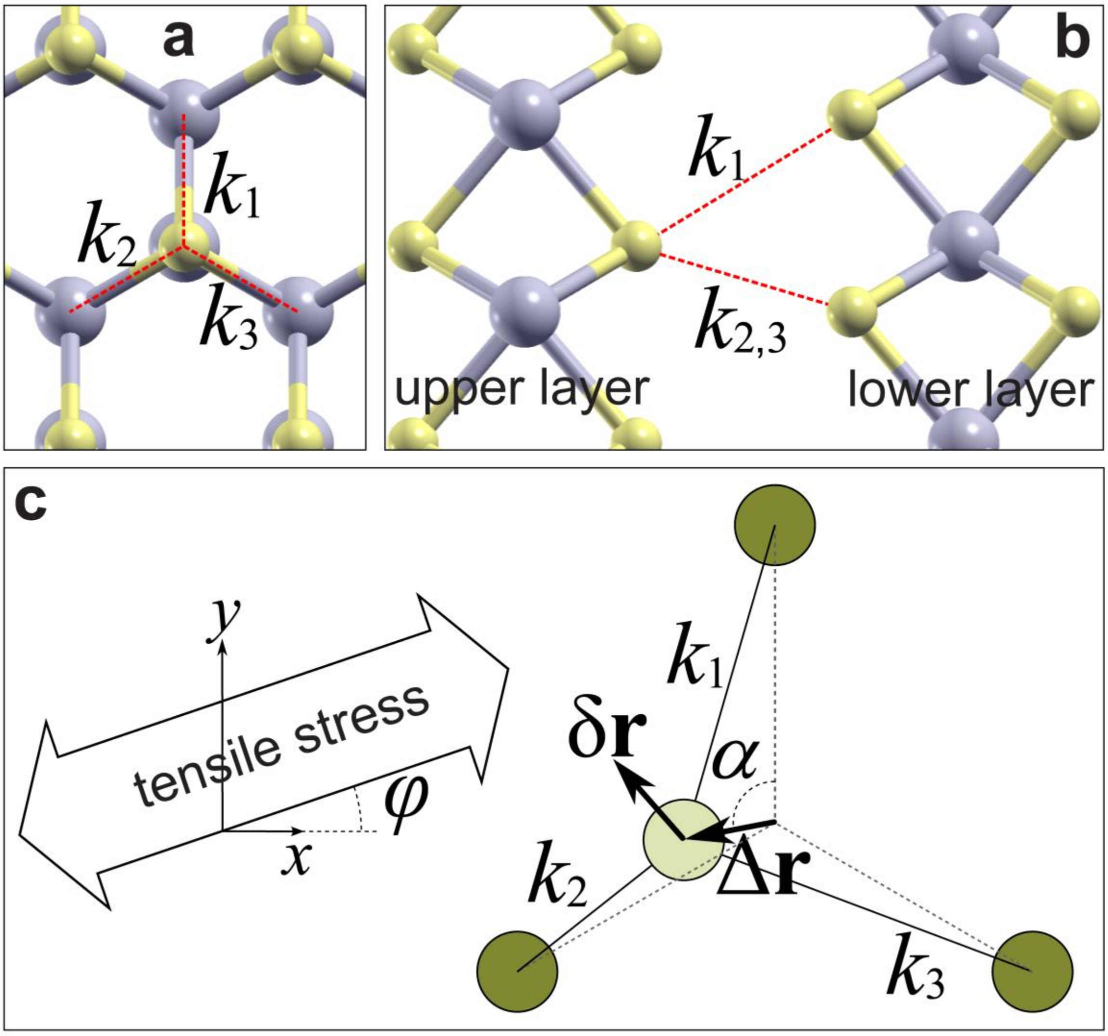


**Supplementary Figure 3| Structure of $MoS_2$ and definition of parameters.** (**a**) Top and (**b**) side view of a relaxed structure of bilayer 2H-$MoS_2$. $k_1$, $k_2$, and $k_3$ are indicated. (**c**) Ball-and-stick model with interlayer sliding.

effective spring constants between one of the sulfur atoms in the upper layer and its three nearest-neighbor sulfur atoms from the lower layer. $\delta\mathbf{r}$ in Supplementary Fig. 3c is the lateral dynamical displacement vector of an interlayer shear phonon mode from the shifted position $\Delta\mathbf{r}$. Let us set the direction of the sliding to be $\alpha$ from the positive *y* axis so that $\alpha = -2\varphi$ for $s_{14} > 0$ and $\alpha = \pi - 2\varphi$ for $s_{14} < 0$. Here, $\Delta\mathbf{r}$ and $\varphi$ are defined in the main text.

The lateral sliding, $\Delta\mathbf{r}$, changes the frequency of the interlayer shear mode by modifying the interatomic spring constants, $k_i$'s. The interatomic spring constant changes linearly if the sliding is small enough so that $k_i = k - \gamma'\Delta a_{\mathrm{SS}}^i$, where *k* is the spring constant without strain, $\gamma'$ is a linear scaling factor, and $\Delta a_{\mathrm{SS}}^i = a_{\mathrm{SS}}^i - a_{\mathrm{SS}}$ is the change of the corresponding nearest-neighbor distance with $a_{\mathrm{SS}}$ being the interatomic distance without strain. Up to the first order of $|\Delta\mathbf{r}|$, $\Delta a_{\mathrm{SS}}^1 \approx -|\Delta\mathbf{r}|\cos\alpha$, $\Delta a_{\mathrm{SS}}^2 \approx |\Delta\mathbf{r}|\cos(\frac{\pi}{3}+\alpha)$, and $\Delta a_{\mathrm{SS}}^3 \approx |\Delta\mathbf{r}|\cos(\frac{\pi}{3}-\alpha)$ so that $k_1 \approx k + \gamma'|\Delta\mathbf{r}|\cos\alpha$, $k_2 \approx k - \gamma'|\Delta\mathbf{r}|\cos(\frac{\pi}{3}+\alpha)$, and $k_3 \approx k - \gamma'|\Delta\mathbf{r}|\cos(\frac{\pi}{3}-\alpha)$.

A dynamical displacement vector under an excitation of an interlayer shear phonon mode, $\delta\mathbf{r} = (\delta x, \delta y)$, changes interatomic distances dynamically from the shifted positions such that $\delta a_1 = -\delta y$, $\delta a_2 = \frac{\sqrt{3}}{2}\delta x + \frac{1}{2}\delta y$, $\delta a_3 = -\frac{\sqrt{3}}{2}\delta x + \frac{1}{2}\delta y$. With modified spring constants $k_i$'s and corresponding $\delta a_{\mathrm{SS}}^i$'s, the potential energy is given by $U = \sum_{i=1}^{3} \frac{1}{2} k_i \delta a_i^2$, which in turn can be expressed in terms of $\delta\mathbf{r}$ as $U = \frac{1}{2}\delta\mathbf{r}^T K \delta\mathbf{r}$, where *K* is a $2 \times 2$ matrix,

$$K = \begin{pmatrix} \frac{3}{2}k - \frac{3}{4}\gamma'|\Delta\mathbf{r}|\cos\alpha & \frac{3}{4}\gamma'|\Delta\mathbf{r}|\sin\alpha \\ \frac{3}{4}\gamma'|\Delta\mathbf{r}|\sin\alpha & \frac{3}{2}k + \frac{3}{4}\gamma'|\Delta\mathbf{r}|\cos\alpha \end{pmatrix}. \tag{1}$$

The eigenvalues, $k_\pm = \frac{3}{2}k \pm \frac{3}{4}\gamma'|\Delta\mathbf{r}| = \frac{3}{2}k \pm \frac{3}{4}d_{\mathrm{int}}|s_{14}|\sigma$, and eigenvectors, $\delta\mathbf{r}_+ = (\sin\frac{\alpha}{2}, \cos\frac{\alpha}{2})$ and $\delta\mathbf{r}_- = (\cos\frac{\alpha}{2}, -\sin\frac{\alpha}{2})$, of *K* represent the effective spring constants and displacement vectors of the corresponding normal modes, respectively.

The vibration directions of the phonon modes, $\alpha_\pm$, are obtained from $\tan\alpha_\pm = \delta y / \delta x$ and given by $\alpha_+ = \pi/2 - \alpha/2$ and $\alpha_- = -\alpha/2$. Using the relation between $\alpha$ and $\varphi$ depending on the sign of $s_{14}$, $\alpha_+ = \varphi + \pi/2$ and $\alpha_- = \varphi$ for $s_{14} > 0$ whereas $\alpha_+ = \varphi$ and $\alpha_- = \varphi - \pi/2$ for $s_{14} < 0$. Since the reduced mass of interlayer shear modes for a primitive unit cell is $m_{\mathrm{r}} = (m_{\mathrm{Mo}} + 2m_{\mathrm{S}})/2$, the phonon frequencies, $\omega_\pm = \sqrt{k_\pm / m_{\mathrm{r}}}$, are

$$\omega_\pm \approx \omega_0 \pm \frac{\omega_0 d_{\mathrm{int}} \gamma' |s_{14}| E_{\mathrm{i}}}{4k} \varepsilon, \qquad (2)$$

where $\omega_0 = \sqrt{3k / (m_{\mathrm{Mo}} + 2m_{\mathrm{S}})}$ and $m_{\mathrm{Mo}}$ and $m_{\mathrm{S}}$ are the masses of molybdenum and sulfur atoms, respectively. Here, we used $\varepsilon = \sigma / E_{\mathrm{i}}$ where $\varepsilon$ is the strain along the applied uniaxial stress $\sigma$, and $E_{\mathrm{i}}$ is the in-plane Young's modulus.

Supplementary Equation (2) reflects only the effect of induced sliding that causes splitting, not the effect of the direct spring constant modification due to the tensile strain which is supposed to shift the average of split frequencies, $\bar{\omega} = \frac{\omega_+ + \omega_-}{2}$. In order to estimate $\bar{\omega}$, we consider the case of an equibiaxial strain, $\varepsilon_{\mathrm{eq}}$, where all the spring constants are scaled as $k \to k - \gamma' a_{\mathrm{SS}} \varepsilon_{\mathrm{eq}}$ so that the effective spring constant for the reduced mass system becomes $\frac{3}{2}k \to \frac{3}{2}k - \frac{3}{2}\gamma' a_{\mathrm{SS}} \varepsilon_{\mathrm{eq}}$. The corresponding shift of the average frequency becomes $\Delta\bar{\omega} = -\frac{\omega_0 \gamma' a_{\mathrm{SS}}}{2k} \varepsilon_{\mathrm{eq}}$ .Using the in-plane Poisson's ratio of the bilayer $MoS_2$, $\nu_{\mathrm{i}} = 0.22$ and

assuming linear response of the sample to the external strain, $\Delta\bar{\omega}$ for our experiment will become

$$\Delta\bar{\omega} = -\frac{(1-\nu_{\mathrm{i}})\omega_0\gamma' a_{\mathrm{SS}}}{4k}\varepsilon \tag{3}$$

It is worth noting that from Supplementary Equations (2) and (3), $|s_{14}|$ can be expressed as

$$|s_{14}| = \frac{a_{\mathrm{SS}}}{d_{\mathrm{int}}}\frac{(1-\nu_{\mathrm{i}})}{2E_{\mathrm{i}}}\frac{(\omega_+ - \omega_-)}{\Delta\bar{\omega}}\,, \tag{4}$$

where $\omega_+ - \omega_-$ and $\Delta\bar{\omega}$ can be determined by Raman measurements.

**Supplementary Note 2: First principles calculations of elasticity constants**

The shear part of the stiffness tensor of a system with $D_{3d}$ symmetry in a matrix form can be written by only three independent parameters,

$$\begin{pmatrix} \sigma_4 \\ \sigma_5 \\ \sigma_6 \end{pmatrix} = \begin{pmatrix} c_{44} & 0 & 0 \\ 0 & c_{44} & c_{56} \\ 0 & c_{56} & c_{66} \end{pmatrix} \begin{pmatrix} \varepsilon_4 \\ \varepsilon_5 \\ \varepsilon_6 \end{pmatrix} \tag{5}$$

with the choice of $z$ as the axis for the three-fold rotational symmetry. The components of stiffness tensor can be obtained by differentiating the total energy $E_{tot}$ in terms of shear strain; $c_{44} = \partial^2 E_{tot} / \partial\varepsilon_4^2$, $c_{66} = \partial^2 E_{tot} / \partial\varepsilon_6^2$, and $c_{56} = \partial^2 E_{tot} / \partial\varepsilon_5 \partial\varepsilon_6$. By taking the inverse of stiffness tensor, one can get the shear part of compliance tensor, Eq. (1) in the main text. Using a first-principles approach based on density-functional theory with plane wave basis set, we calculate total energies $E_{tot}(\varepsilon_4, \varepsilon_5, \varepsilon_6)$, at 5×5×5 grid points in the shear strain space.

For the total energy calculations with tensile strain for a bilayer system, in-plane primitive unit cell vectors $u_1 = (a,0,0)$ and $u_2(b,c,0)$ are used. The unit cell along the $z$ axis is set to be larger than 7 nm in order to reduce interactions between supercells. Let us define $a_0$, $b_0$, $c_0$ for the values of $a$, $b$, $c$ for a system without strain, which has relations, $b_0 = a_0 / 2$ and $c_0 = \sqrt{3} a_0 / 2$. For simulating interlayer shear strain, we further define the displacement of the top layer with respect to the bottom layer along the $x$ and $y$ directions as $disp_i$, where $i = x, y$. Inter- and intra-layer shear strains are defined as $\varepsilon_4 = 2\varepsilon_{yz} = disp_y / d_{int}$, $\varepsilon_5 = 2\varepsilon_{xz} = disp_x / d_{int}$, and $\varepsilon_6 = 2\varepsilon_{xy} = (b - b_0) / c_0$. Here, $d_{int}$ is the interlayer distance. The values for $c_{44}$, $c_{66}$, and $c_{56}$ are calculated by interpolating the total energy in the shear strain grid and so is the compliance tensor.

**Supplementary Note 3: Calculation of Grüneisen parameters**

Assuming linear dependence of the frequencies on the uniaxial strain, the shift rates of the $E_g^-$, $E_g^+$, and $A_{1g}$ modes for bilayer $MoS_2$ are obtained to be $-4.0 \pm 0.1$, $-0.9 \pm 0.2$, and $-0.3 \pm 0.1$ $cm^{-1}\%^{-1}$, respectively. From the shift rates, the Grüneisen parameter ($\gamma$) and the deformation potential ($\beta$) were calculated for the $E_g$ mode of bilayer $MoS_2$ using the following equations[1].

$$\gamma = \frac{\Delta\omega_+ + \Delta\omega_-}{2\omega_0(1-\nu_i)\varepsilon} \quad \text{and} \tag{6}$$

$$\beta = \frac{\Delta\omega_+ - \Delta\omega_-}{2\omega_0(1+\nu_i)\varepsilon}, \tag{7}$$

where $\omega_0$ is the peak position of the $E_g$ mode without strain, $\Delta\omega_{+(-)}$ is the peak shift of $E_g^+$ ($E_g^-$) under strain with respect to $\omega_0$, $\varepsilon$ is the strain, and $\nu_i$ is the in-plane Poisson's ratio of $MoS_2$. We used the Poisson's ratio of 0.22[2]. The obtained parameters are $\gamma = 1.0 \pm 0.2$ and $\beta = 0.8 \pm 0.2$ for the $E_g$ mode. We applied the same method to the interlayer shear (*S*) mode and obtained $\gamma = 0.9 \pm 0.3$ and $\beta = 2.4 \pm 0.4$.

**Supplementary Note 4: Determination of crystallographic orientation**

For the $E_g^-$ mode, the atomic displacement is parallel to strain axis, whereas the $E_g^+$ mode is perpendicular to it. By considering a linear combination of the two modes, one can obtain the polarization dependence of $E_g^-$ and $E_g^+$ modes as follows[1].

$$I_{E_g^-} \propto \sin^2(\theta_i + \theta_s - 3\varphi) \quad \text{and} \quad I_{E_g^+} \propto \cos^2(\theta_i + \theta_s - 3\varphi), \qquad (8)$$

where $\theta_i$ is an angle between the incident polarization and the strain direction, $\theta_s$ is an angle between the scattered polarization and the strain direction, and $\varphi$ is an angle between the strain direction and the zigzag direction as shown in Fig. 1a. By fitting the experimental data with the above equations, we obtained the crystallographic orientation of the samples with respect to the zigzag direction.

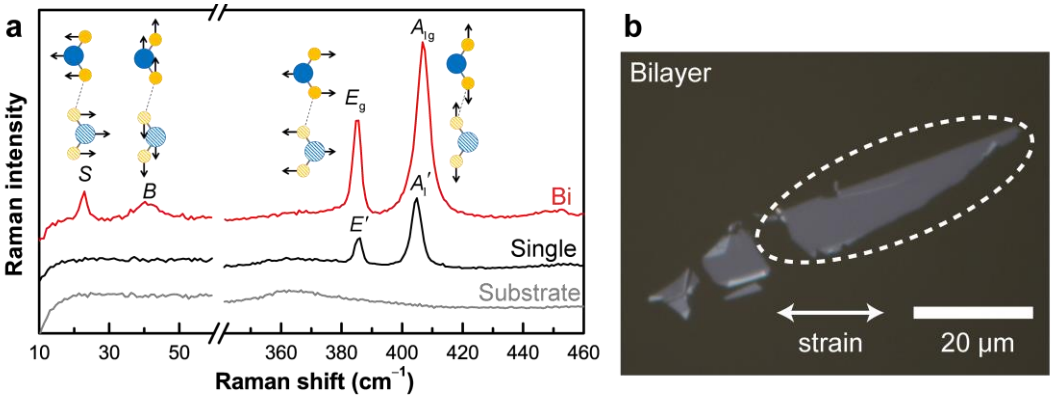


**Supplementary Figure 4| Raman spectra and optical image of samples.** (**a**) Raman spectra of single and bilayer $MoS_2$ on acrylic substrate. (**b**) Optical image of bilayer sample.

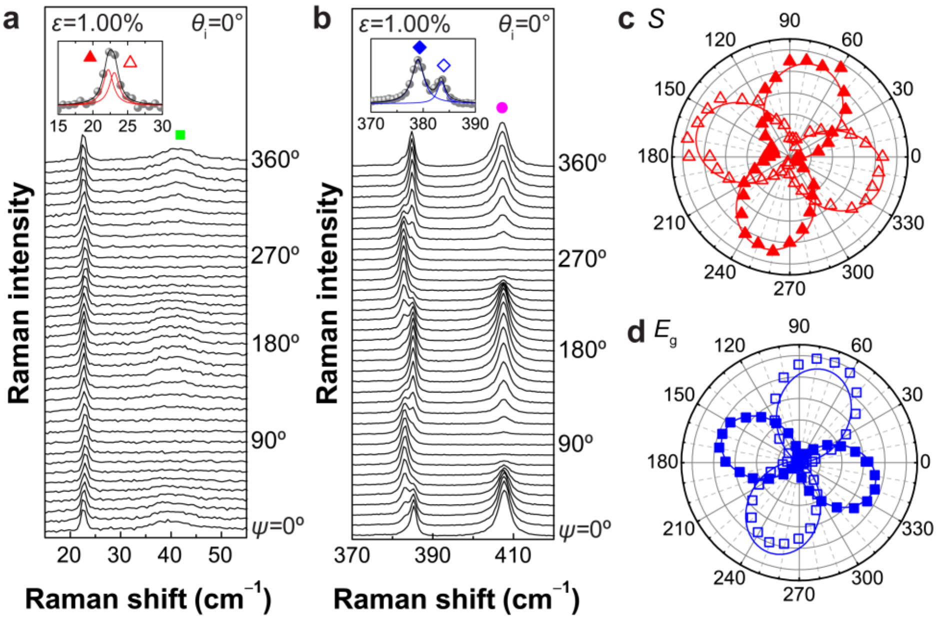


**Supplementary Figure 5| Polarization dependence of Raman modes for an additional sample**. Polarized Raman spectra of strained bilayer $MoS_2$ ($\varepsilon = 1.00\%$) for (**a**) inter- and (**b**) intra-layer modes. The incident polarization ($\theta_i$) is fixed at 0° and the spectra are measured as a function of $\psi$ defined in Fig. 1a. Symbols are defined in Fig. 2. Normalized polar plots of (**c**) $E_g$ ($E_g^-$ and $E_g^+$) and (**d**) shear ($S$, $S^-$ and $S^+$) modes.

**Supplementary References**


1. Mohiuddin, T. M. G. *et al.* Uniaxial strain in graphene by Raman spectroscopy: *G* peak splitting, Grüneisen parameters, and sample orientation. *Phys. Rev. B* **79,** 205433 (2009).
2. Woo, S., Park, H. C. & Son, Y.-W. Poisson's ratio in layered two-dimensional crystals. *Phys. Rev. B* **93,** 75420 (2016).
